# Size Polymorphism in Alleles of the Myoglobin Gene from *Biomphalaria* Mollusks

**DOI:** 10.3390/genes1030357

**Published:** 2010-10-20

**Authors:** Kádima N. Teixeira, Karyne N. Souza, Teofânia H.D.A. Vidigal, Cristiane A. Brito, Alexandre M.C. Santos, Marcelo M. Santoro

**Affiliations:** 1 Department of Biochemistry, Immunology, Federal University of Minas Gerais, Belo Horizonte/MG, 31270-901, Brazil; E-Mails: alexandremcs@ccs.ufes.br (A.M.C.S.); santoro@icb.ufmg.br (M.M.S.); 2 Department of Zoology, Federal University of Minas Gerais, Belo Horizonte/MG, 31270-901, Brazil; E-Mails: knsouza@hotmail.com (K.N.S.); teofania@icb.ufmg.br (T.H.D.A.V.); 3 Research Center René Rachou, Belo Horizonte/MG, 30190-002, Brazil; E-Mail: britoc@cpqrr.com.br; 4 Department of Physiological Sciences, Federal University of Espírito Santo, Vitória/ES, 29075-91, Brazil

**Keywords:** *Biomphalaria*, myoglobin gene, size polymorphism

## Abstract

Introns are common among all eukaryotes, while only a limited number of introns are found in prokaryotes. Globin, globin-like proteins are widely distributed in nature, being found even in prokaryotes, a wide range of patterns of intron-exon have been reported in several eukaryotic globin genes. Globin genes in invertebrates show considerable variation in the positions of introns; globins can be found without introns, with only one intron or with three introns in different positions. In this work we analyzed the introns in the myoglobin gene from *Biomphalaria glabrata*,* B. straminea, B. tenagophila.* In the *Biomphalaria* genus, the myoglobin gene has three introns; these were amplified by PCR, analyzed by PCR-RFLP. Results showed that the size (number or nucleotides), the nucleotide sequence of the coding gene of the myoglobin are variable in the three species. We observed the presence of size polymorphisms in intron 2, 3; this characterizes a homozygous/heterozygous profile, it indicates the existence of two alleles which are different in size in each species of *Biomphalaria*. This polymorphism could be explored for specific identification of *Biomphalaria* individuals.

## 1. Introduction

In 1977, several research groups published their findings about DNA coding sequences (exons) broken by non-coding sequences (introns) in eukaryotes [1,2,3]. Introns are widespread among eukaryotes [4,5], while only a limited number of introns are found in prokaryotes.

Cavalier-Smith [6] suggested that introns were selfish DNA with no distinct cellular function, however there is much to discuss about the presence of these non-coding sequences [7]. After the ENCODE project consortium, it is known that intronic regions are transcribed in humans, indicating that protein coding loci are more transcriptionally complex than previously thought [8,9].

Independently in 1978, Doolittle [10], Darnell [11] proposed the “Introns-Early theory” which states that introns are very early elements in the genome, which have been present since the eukaryote/prokaryote divergence. In the “Introns-Late theory” introns appeared recently in the eukaryotic genome [6,12,13]. A growing collection of introns has been found in different positions in some animal species while closely related species do not have the introns [14,15,16,17,18].

In the evolutionary history of introns, the selective stretch which modulates their evolution, the relative contribution of its eukaryotic gene loss or insertion is not understood [19]. In interpreting the evidence that a few introns may have a common position between animals, plants, fungi, one must bear in mind their evolutionary history. Comparisons of rRNA sequences support the idea that the three kingdoms of multicellular eukaryotes form monophyletic groups, which seem to have originated almost simultaneously, possibly from a common ancestor. This would also explain the conservation of the position of some introns among homologous genes within these kingdoms, without having to invoke the presence of introns in these genes from the very beginning [20]. 

Many authors have attempted to interpret the distribution of intron in terms of a mixture of movement, removal of introns, but the difficulty of phase-shifting movement causes serious doubts about these interpretations. In contrast, some gene families for which phylogenies can be traced show patterns that clearly indicate intron insertions [21].

The protein coding sequences are interrupted by introns at specific points, which could be used to investigate, for instance, the evolution of protein families. The gene encoding the ancestral globin chain is assumed to be interrupted by three introns inserted in the B, E, G helices. However, the conservation of this intron pattern, the exact insertion positions of the introns during evolution is a matter of ongoing discussion [22].

Intron location, sequence may also shed light on the origin of some protein genes, for instance the polymeric globin genes occurring in mollusks, arthropods [23]. In this way introns could be used to study evolution as well as the origin, phylogeny, systematic of the Mollusca phylum. The mollusk Biomphalaria glabrata has three introns in the myoglobin gene at positions A3.2, B12.2, G7.0; the two last ones are conserved.

Identifying individuals from the Biomphalaria genus is a difficult task because they are very similar in morphology. Classic methodology of identification is based on comparative morphology of shells, anatomy of the reproductive organs, however there is much intra-specific variation [24]. Classic methods of identifying Biomphalaria species are important; however association with molecular techniques can result in more solid diagnostic [25]. In 1996, Vidigal, co-workers [26] showed that it is possible to distinguish B. glabrata, B. tenagophila by ribosomal gene amplification. In 1991, Knight, co-workers [27] showed intra-specific variability in B. glabrata susceptibility, resistance to Schistosoma mansoni by RFLP. These results show the efficiency of molecular techniques to identify the mollusks.

In this work, we analyze the introns of the myoglobin gene of mollusks from the Biomphalaria genus in order to evaluate their potential use for precise identification in systematic studies. 

## 2. Results, Discussion

**Figure 1:**
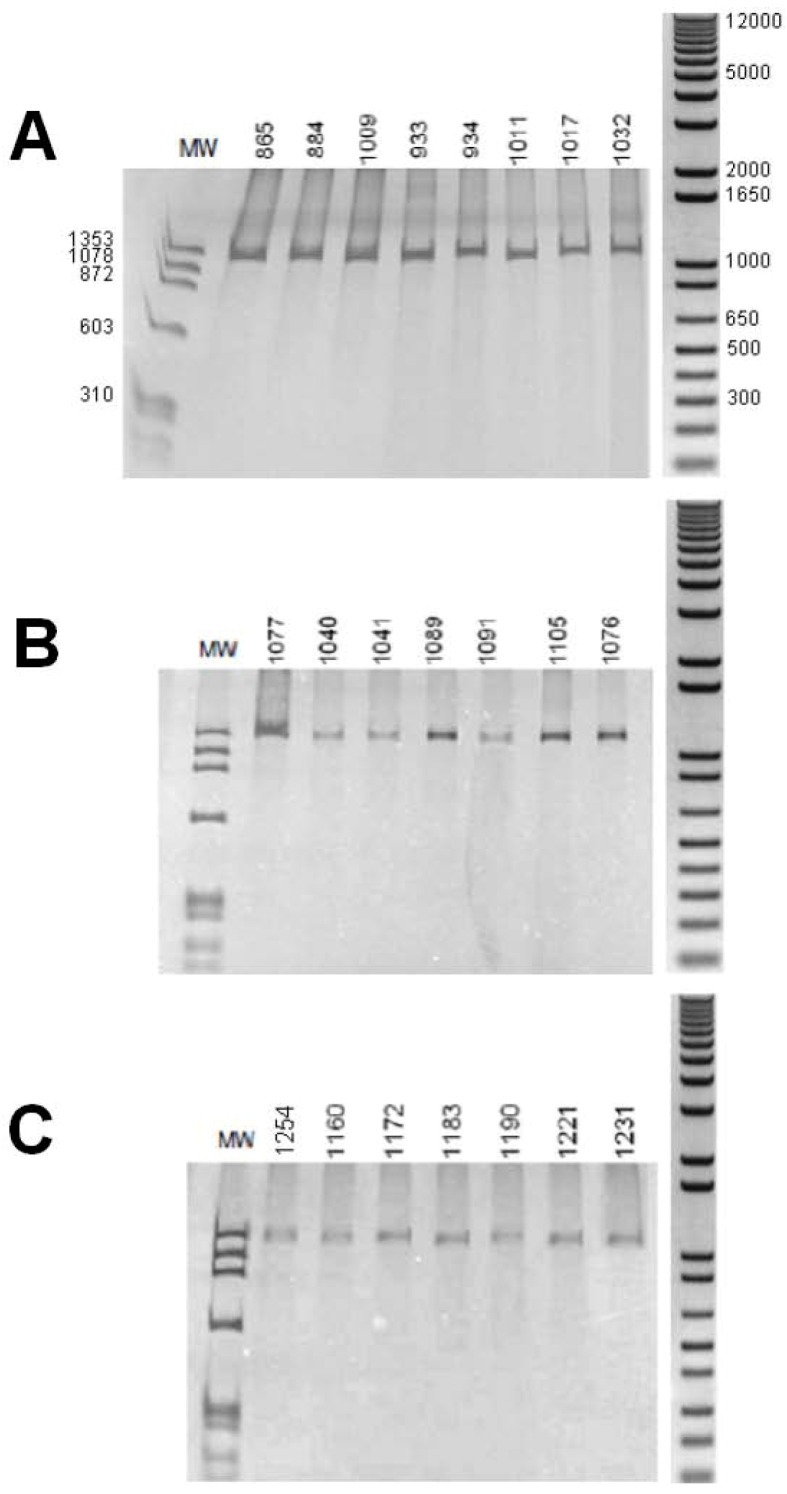
Polyacrylamide gel electrophoresis with silver stain. Amplification of intron 1 of the myoglobin gene. (A) B. glabrata; (B) B. straminea; (C) B. tenagophila. Numbers above the lane are one different individual. MW = molecular weight (base pairs) of φX174/Hae III digested (Amersham Biosciences). On right: molecular weight (base pairs) of 1 kb Plus DNA ladder (Invitrogen).

The size (in base pairs) of the amplicons of intron 1 was analyzed by polyacrylamide gel electrophoresis. All individuals of the three Biomphalaria species showed a DNA fragment of about 1,300 bp (Figure 1).

**Figure 2:**
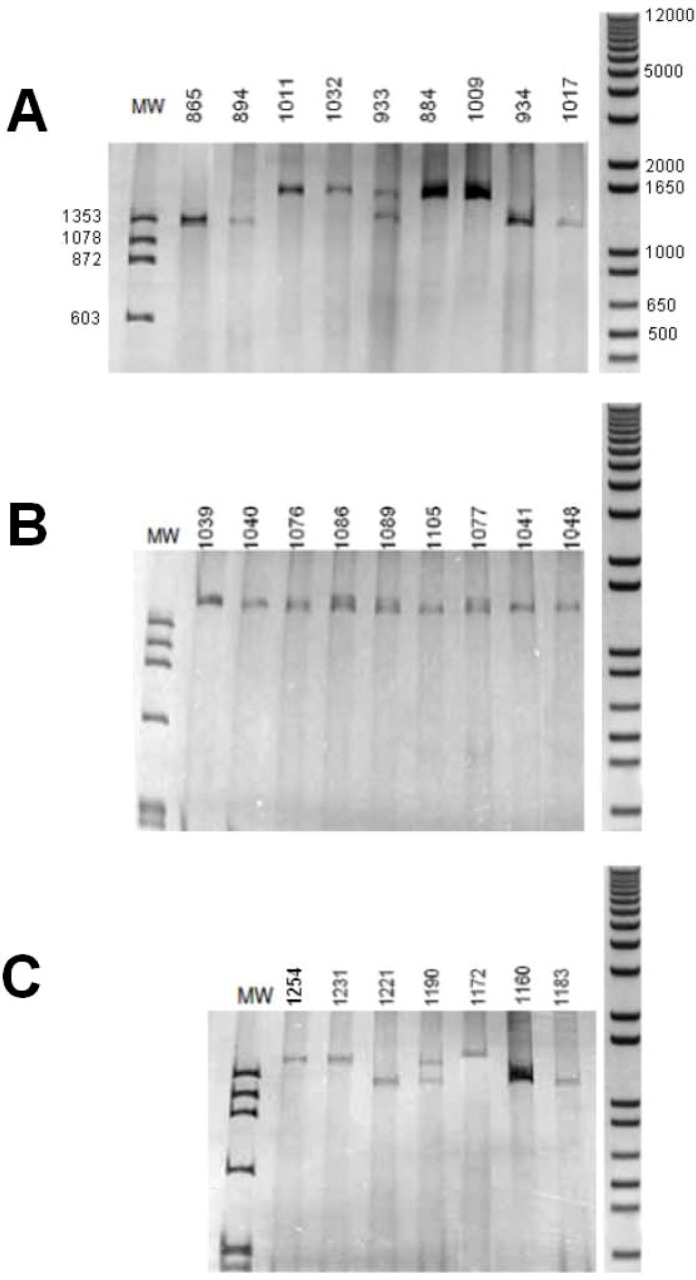
Polyacrylamide gel electrophoresis with silver stain. Amplification of intron 2 of the myoglobin gene. (A) B. glabrata; (B) B. straminea; (C) B. tenagophila. Numbers above the lanes indicate one different individual. MW = molecular weight (base pairs) of φX174/Hae III digested (Amersham Biosciences). Right: molecular weight (base pairs) of 1 kb Plus DNA ladder (Invitrogen).

Amplification of intron 2 of B. straminea generated an amplicon larger than 1,300 bp, of about 1,500 bp. In B. glabrata, B. tenagophila, some individuals were observed to have a large amplicon of about 1,400 bp,, some individuals showed a smaller fragment (of about 1,300 bp for B. glabrata, about 1,200 bp for B. tenagophila), whereas some individuals showed the two fragments (Figure 2).

**Figure 3:**
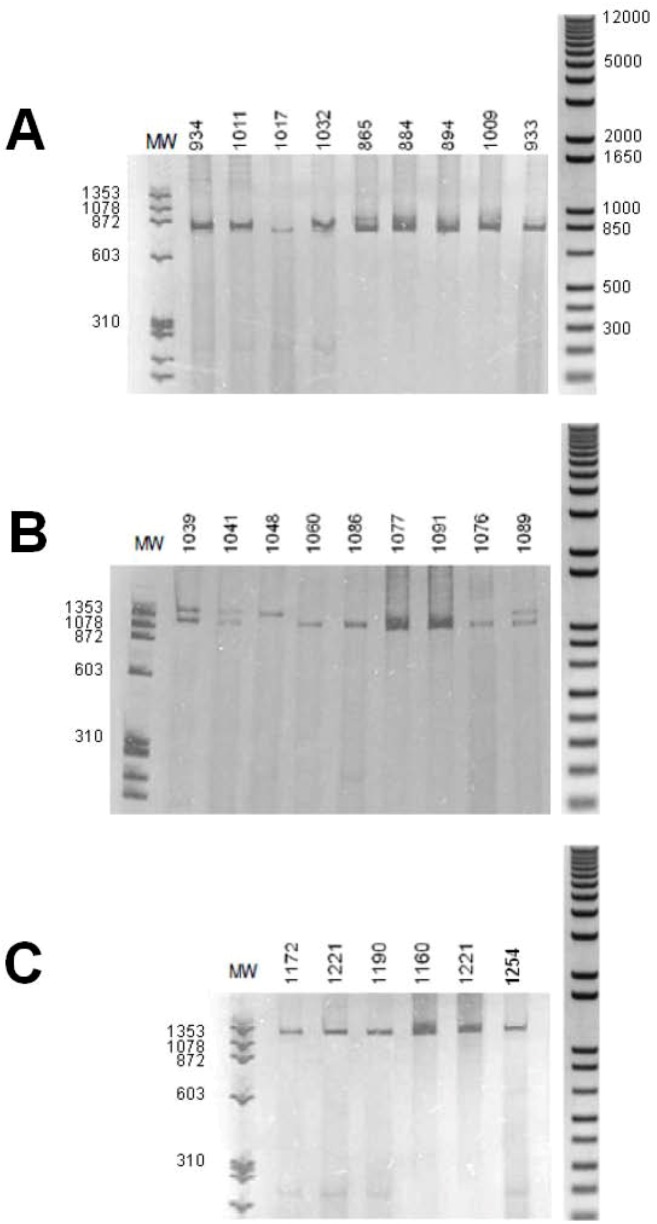
Polyacrylamide gel electrophoresis with silver stain. Amplification of intron 3 of the myoglobin gene. (A) B. glabrata; (B) B. straminea; (C) B. tenagophila. Numbers above the lane are one different individual. MW = molecular weight (base pairs) of φX174/Hae III digested (Amersham Biosciences). Right: molecular weight (base pairs) of 1 kb Plus DNA ladder (Invitrogen).

The amplification of the intron 3 presented a similar homozygous/heterozygous pattern in B. straminea. Some individuals showed an amplicon of about 1,400 bp, other individuals showed an amplicon of about 1,200 bp,, some ones showed the two amplicons. In B. glabrata, was observed one amplicon only of about 750–850 bp,, B. tenagophila showed an amplicon of about 1,300 bp (Figure 3). 

Clear, satisfactory amplification using genomic DNA as a template is not a simple task, since nonspecific amplifications, artifacts could contaminate the true results. After obtaining the best protocol for amplification, this was repeated at least five times. The monitoring of all amplifications showed the results on the size of introns from Biomphalaria, which are described in the text; the gels presented as figures have some artifacts that do not refer to amplification of introns.

**Figure 4:**
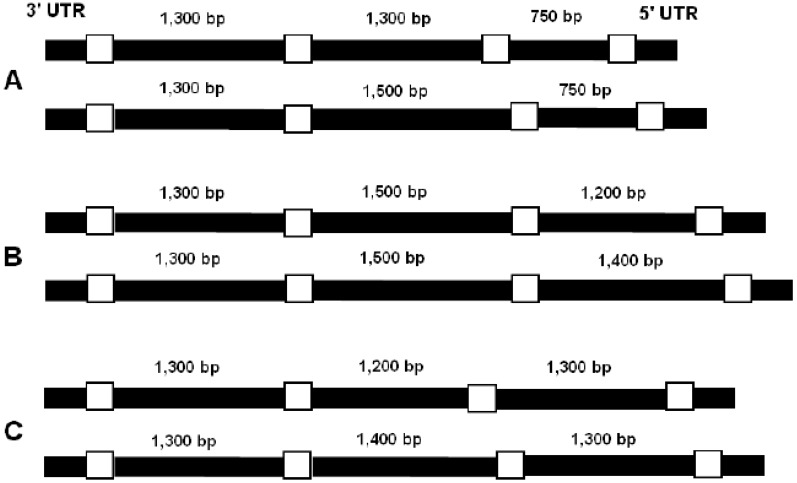
Schematic representation of the supposed alleles from (A) B. glabrata, (B) B. straminea,, (C) B. tenagophila according to amplicons obtained. Exons (white) were represented only to delimit introns (black).

The models for putative alleles from the three Biomphalaria species are shown in Figure 4. They were built based on the nucleotide sequence of the myoglobin gene from B. glabrata deposited in data bank, sequence of the cDNA myoglobins of B. tenagophila, B. straminea that were deposited in Genbank (accession nº EF646378, EF646379), which are very similar to B. glabrata myoglobin (>96% identity). 

PCR-RFLP was used to obtain rapid information about the nucleotide sequence of the myoglobin introns from Biomphalaria; for individuals who had introns of different sizes, the largest fragment was digested.

**Figure 5:**
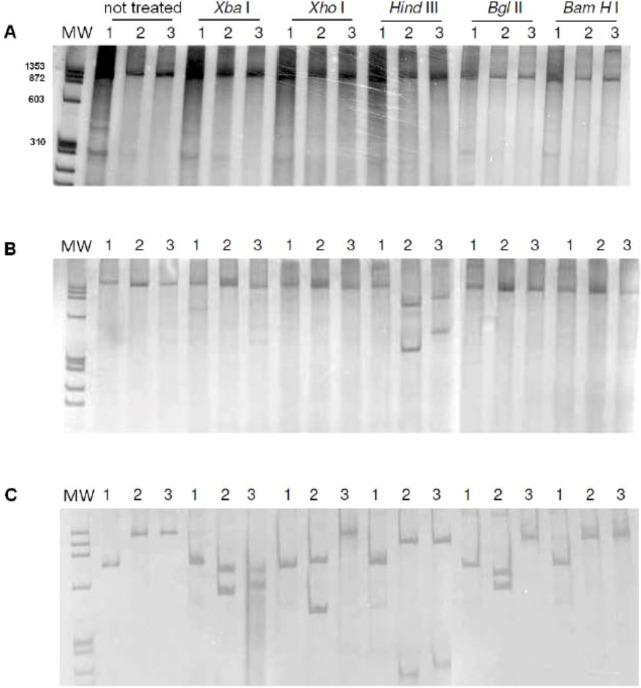
Polyacrylamide gel electrophoresis with silver stain. PCR-RFLP of the amplicons of the introns from Biomphalaria myoglobin. 1. B. glabrata; 2. B. straminea; 3. B. tenagophila. MW = molecular weight (base pairs) of φX174/Hae III (Amersham Biosciences). Above the lanes, restriction endonucleases are indicated.

In no individual from the Biomphalaria species was fragmentation of intron 1 observed by restriction enzymes. The intron 2 from B. straminea, B. tenagophila was digested by Hind III only, however it was digested in different places, generating fragments of different sizes.

Intron 3 from B. straminea was cutted by Xba I, Xho I, Bgl II, Hind III,, the same intron from B. tenagophila was cutted by Xba I, Hind III. Intron 3 from B. glabrata was not cut by any enzyme (Figure 5).

In 2004, Sokolova, Boulding [28] described size polymorphism in one intron of the enzyme N-aminopeptidase among individual mollusks of the Littorina genus. A similar result has been obtained in this work in the myoglobin gene of the three species of Biomphalaria mollusks. We observed that B. tenagophila, B. glabrata, B. straminea have size polymorphism in the myoglobin gene. This polymorphism, which characterizes a profile of homozygosity/heterozygosity for the introns of the myoglobin gene, had not been described for B. glabrata, whose gene has been characterized.

The first intron of B. glabrata has 1,116 bp, the second intron has 1,008 bp, third has 582 bp [18]. The difference in size observed between the cited introns of B. glabrata, those obtained in this work is due to segments of exons and/or segments of the UTR amplified together with the intron in the PCR. The same referred fragments are present in the introns of the other Biomphalaria species analyzed.

The first elucidation of globin gene structure was that of the human hemoglobin α, β chains. These genes have two introns conserved exactly at positions B12.2 (α-helix B in the protein), G6.3 [29]. According to Dewilde et al. [22], the structure of the globin gene of B. glabrata contains three intron/four exon. The B. glabrata myoglobin gene contains an unprecedented α-helix intron at position A3.2 that encodes a monodomain globin. It must, therefore, be considered as a newly inserted intron. The analyses of the amplicons of the introns from B. tenagophila, B. straminea suggest the same pattern for myoglobin gene. 

The typical gene structure of three exons/two introns are found in all vertebrate globins, in most annelids [30]. The results observed in amplicons from intron 2, 3 suggest that the studies of Biomphalaria species present a homozygous/heterozygous pattern in the second, third intron of the myoglobin gene which can be observed by its size. The polymorphisms are confined to introns 2, 3. Perhaps, it is possible that the conservation of size in intron 1 is because this intron position is evolutionarily most recent.

The globin genes of vertebrates, plants contain, respectively, two, three introns, their insertion positions are conserved. The three intron/four exon pattern of plants is proposed to be ancestral, all other globin gene structures would be derived mainly by intron loss [31]. Several non vertebrate, protozoan globin genes have been characterized, it has become clear that the intron/exon pattern is less conserved than originally expected [32]. 

B. straminea presents a size polymorphism in the third intron of the myoglobin gene. B. tenagophila, B. glabrata contain size polymorphism in the second intron,, any of the three species showed size polymorphism in the first intron. This kind of polymorphism, the homozygous/heterozygous pattern was not shown for the myoglobin gene of B. glabrata which had been described.

Analysis of unique individuals indicates that there are two alleles of different sizes which code for myoglobins in the Biomphalaria genus. The difference is about 200 bp, each Biomphalaria specie presents polymorphism in one intron only. The smaller alleles were observed in B. glabrata, the bigger ones were observed in B. straminea. By analyzing the size it is believed that the nucleotide sequence of the myoglobin gene of the B. glabrata deposited in data bank could be the minor allele of this specie.

No differences were observed in size of the primary sequences (amino acids) of the myoglobins from the three Biomphalaria species such that the contribution of the exon to the size of the amplicons is the same for all three Biomphalaria species. The difference among the myoglobin introns from B. glabrata, B. straminea e B. tenagophila suggests a potential for using these as molecular markers to carry out the specific identification of these mollusks. 

Analysis of the distribution of the possible alleles of the gene encoding myoglobin among individuals of three species of Biomphalaria do not indicated a relation to the geographical origin of the mollusks, but this relationship cannot be ruled out, since it would be necessary to analyze a larger number of individuals.

The sequencing of heterozygous genotypes with different alleles in size is a difficult task,, RFLP analysis is a way to get quick information about the nucleotide sequence [28]. Analysis by PCR-RFLP of myoglobin introns showed that introns 2, 3 are different in the three Biomphalaria species. Intron 1 was not cut by any of the five enzymes chosen, but this does not mean that the nucleotide sequence is the same, since introns are elements that accumulate a large number of mutations during evolution [7].

The Biomphalaria genus covers organisms morphologically very similar, making their identification difficult. The classical identification is based on comparative morphology of the shells, anatomy of reproductive organs, but the enormous intraspecific variation of morphological, anatomical features commits to a specific classification. Identification may also be hampered by the small size of the specimens, whose distinctive morphological characters are not very evident [24].

Monis [25] highlights the importance of traditional methods of identification,, suggests its association with molecular techniques for obtaining a more robust, reliable result. In 1996, Vidigal, co-workers [26] demonstrated that it is possible to specifically differentiate B. glabrata, B. tenagophila by amplification of portions of their ribosomal gene. These same authors in 2001 were able to separate the species B. glabrata, B. tenagophila, B. straminea by PCR-RFLP using the gene for ITS (internal transcribed spacer) that refers to a piece of non-functional RNA situated between structural ribosomal RNA.

In the same context, Knight et al. [27], using the gene for ribosomal RNA to detect size polymorphisms in restriction fragments (RFLP), were able to show intraspecific variation in B. glabrata susceptible, resistant to infection by Schistosoma mansoni. These results show the efficiency of molecular techniques in the identification of these mollusks.

## 3. Experimental Section

### 3.1. Snails

B. glabrata, B. tenagophila, B. straminea from several Brazilian provinces (Table 1) belonging to Teofânia H.D.A. Vidigal’s (Laboratório de Malacologia e Sistemática Molecular/ICB/UFMG) collection were used. These individuals were collected in their habitat in the environment, thus there was no genetic cross between them. Every individual was analyzed in regard to infection by Schistosoma mansoni. After a negative result for infection, fragments from foot tissue were frozen at -80 °C. 

**Table 1:** Individuals from Biomphalaria genus distributed according to Brazilian province.

Species	Individuals a	Province from Brazil
B. glabrata	865	South East
	884, 1009, 1011, 1017	North East
	933, 934, 1032	North
B. straminea	1040, 1041,	North
	1076, 1077, 1091	North East
	1089, 1105	South East
B. tenagophila	1160, 1183, 1231	South East
	1172, 1190	South
	1221	North East
	1254	Central West

a Number of the individual in the collection from Vidigal, T.H.D.A.

### 3.2. Genomic DNA Extraction of Snails

Genomic DNA was purified from foot tissue of each individual of Biomphalaria using a DNA purification kit (Wizard Genomic DNA Purification, Promega) according to recommendations from the manufacturer. The DNA quantification, purity were analyzed by spectrophotometry at 260, 280 nm using a UV-160A UV-Visible recording spectrophotometer (Shimadzu). The DNA quality was determined by 1% agarose gel electrophoresis. The DNA was not pooled at any step.

### 3.3. Amplification of the Introns from the Biomphalaria Myoglobin Gene 

**Figure 6:**
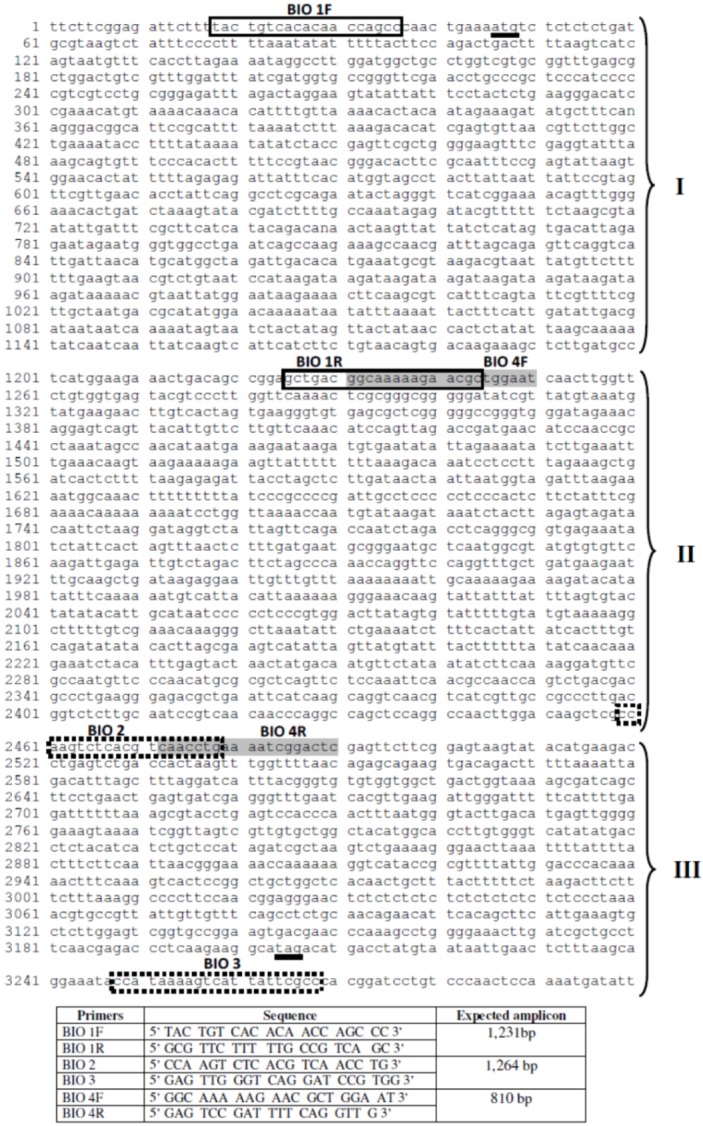
Annealing position of the primers related to the myoglobin gene from B. glabrata (U89283.1). Solid line: Primers BIO 1F/R. Dashed line: BIO 4F/R. Grey box: BIO2/3. Tick line: Initiation, stop codon. I, II, III: introns 1, 2, 3 respectively. Below the figure, the sequence of the primers, expected sizes of amplicons according to the gene U89283.1 are shown.

Amplification of the introns were performed by PCR using three sets of primers which were drafted based on nucleotide sequence from the B. glabrata myoglobin gene available in GenBank Accession Code U89283. The primers were BIO 1F, BIO 2 (Dewilde et al. 1998), the other primers annealing in exon regions or in the UTR (untranslated region) to provide the complete sequence of the three introns of the myoglobin gene (Figure 6). All amplifications were reproduced at least five times.

Primer pair BIO 1F (forward) (5’ TACTGTCACACAACCAGCCC 3’), BIO 1R (reverse) (5’ GCGTTCTTTTTGCCGTCAGC 3’) were used to amplify the first intron (intron 1), primers BIO 4F (5’ GGCAAAAAGAACGCTGGAAT 3’), BIO 4R (5’ GAGTCCGATTTTCAGGTTG 3’) were used to amplify the second intron (intron 2), primers BIO 2, BIO 3 (5’ GAGTTGGTCAGGATCCGTGG 3’) were used to amplify the third intron (intron 3).

The PCR for intron 1 was performed for 30 cycles of 94 °C for 30 s, 65 °C for 60 s, 72 °C for 2 min, a final elongation step at 72 °C for 10 min. The amplification of the introns 2, 3 used the same reaction, except for an annealing temperature of 60 °C. The amplicons were analyzed by 6% polyacrylamide gel electrophoresis with silver stain; the base pair markers φX174/Hae III digested (Amersham Biosciences), 1 kb Plus DNA ladder (Invitrogen) were applied.

### 3.4. PCR-RFLP

PCR-RFLP was used to analyze the similarity of the nucleotide sequence from myoglobin introns. The three introns of the myoglobin gene from three Biomphalaria species were amplified as previously described, amplicons were submitted to digestion with the restriction endonucleases Xba I, Bgl II, Xho I, Hind III, Bam HI (Invitrogen). Only intronic fragments were digested, because the aim was to observe if the nucleotide sequence of the introns differed among species of Biomphalaria. The use of the full gene (whole gene amplification) in RFLP would not allow identification of the differences (cuts) belonging to the introns, exons,, in which intron. Reactions were performed according to the manufacturer, were evaluated by 6% polyacrylamide gel electrophoresis with silver staining; the base pair marker φX174/Hae III digested (Amersham Biosciences) was applied. 

## 4. Conclusion

The gene encoding myoglobin from B. glabrata, B. straminea, B. tenagophila displays size polymorphisms (base pairs) in the intronic regions, featuring a profile of homozygosity/heterozygosity in this gene. Considering the difference among the introns of myoglobin from B. glabrata, B. straminea, B. tenagophila, there is the possibility of using these introns as molecular markers for specific identification of these mollusks.
